# Case 6/2019 - Total Atrioventricular Septal Defect, 12 Years After Operative Correction, No Residual Defects

**DOI:** 10.5935/abc.20190192

**Published:** 2019-11

**Authors:** Edmar Atik, Maria Angélica Binotto, Alessandra Costa Barreto

**Affiliations:** 1 Instituto do Coração do Hospital das Clínicas da Faculdade de Medicina da Universidade de São Paulo, São Paulo, SP - Brazil

**Keywords:** Heart defects, Congenital/surgery, Heart Septal Defects, Ventricular, Postoperative evolution

## Clinical Data

A 14-year-old patient was asymptomatic in all routine postoperative evaluations and in full physical activity, compared to others. During this period, which began when the patient was 21 months old, due to the type A atrioventricular (AV) septal defect totally corrected, no cardiac murmur was heard, the cardiac area remained normal and did not require the use of specific medication. An Echocardiography showed no residual defects, normal heart cavities and no valve regurgitation. The defect was diagnosed from birth and follow-up before the surgery was marked by obvious signs of heart failure due to the volume overload imposed by valve insufficiency and intercavity communications.

**Physical exam:** good general conditions, eupneic, acyanotic, normal 4-limb pulses. Weight: 30 Kg, Height: 140 cm, BP: 105 x 60 mmHg, HR = 76 bpm, O_2_ Sat = 95%.

**Precordium:** *non-palpated ictus* cordis, without systolic impulses. Normophonetic heart sounds, with the second constant sound unfolding. No audible heart murmurs. Liver not palpated and lungs cleared.

### Complementary tests

**Figure 1:**
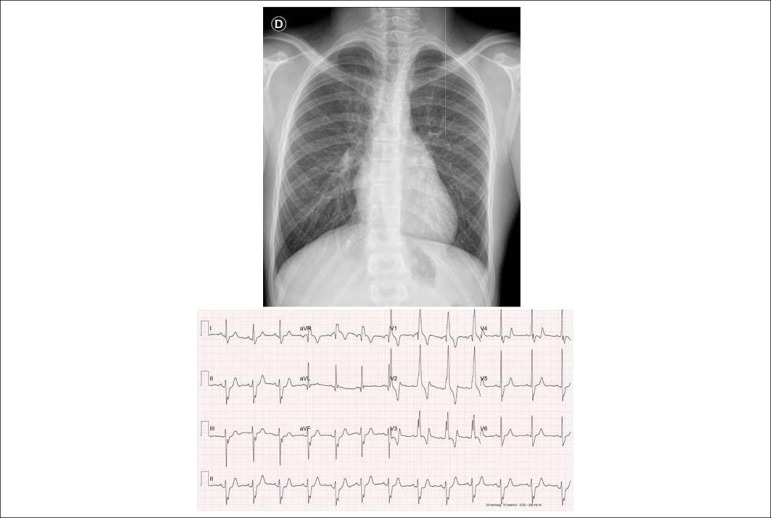
Chest radiography highlights the cardiac area and pulmonary vascular network within normal limits. The middle arch is retified, and the electrocardiogram shows signs of a slight final conduction disorder at the right branch.

**Electrocardiogram:** Sinus rhythm with complete right bundle branch block associated with left anterior superior divisional block with enlarged QRS. Ventricular repolarization is normal. AP = +30^o^, AQRS = -70^o^, AT+ + 60^o^. (Figure 1).

**Chest radiography:** Normal cardiac area (CTI = 0.44) and also normal pulmonary vascular network. The middle arch is rectified and the aortic knob slightly protruding (Figure 1).

**Figure 2:**
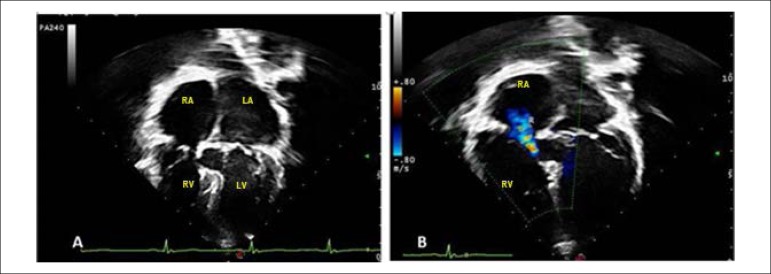
Echocardiogram shows a 4-chamber view of a subcostal section with normal-sized cardiac cavities, with closed defects and good atrioventricular valve coaptation, in A and B. RA: right atrium; LA: left atrium; RV: right ventricle; LV: left ventricle.

**Echocardiography**: Normal AV connection with interatrial and interventricular septa closed by patches and AV valves do not present regurgitation. The right valve had a maximum residual gradient of 6 mmHg and a mean 2 mmHg residual gradient and the left valve was 11 and 2.5 mmHg, respectively. The right ventricular systolic pressure was 27 mmHg. The cardiac cavities had normal sizes and normal left ventricular function (Figure 2).

**Preoperative echocardiogram** *:* situs solitus levocardia. AV connection through single valve with borders inserted into the top of the ventricular septum (Rastelli type A) with mild bilateral insufficiency. *Ostium primum* atrial septal and interventricular input communication were 21 mm in length, causing a marked dilated heart, especially in the right cavities.

**Preoperative cardiac catheterization**: PVR = 1,9 UW, SVR = 12,3 UW, PVR/SVR = 0,15. Ventricular pressures were equalized as well as atrial pressures and pulse pressure was increased in the pulmonary artery tree.

**Clinical diagnosis:** Total defect of the AV septum with extensive intercavity, atrial and ventricular communications, corrected at 21 months of age, with good evolution until the patient was 14 years old, no symptoms and no residual defects, functionally compared with normal people with the same age.

**Clinical reasoning:** The absence of symptoms and normal cardiovascular semiology in patients with previously corrected cardiac structural defects highlights the absence of residual defects due to their adequate correction in all cases. Such condition is usual in acyanogenic congenital defects such as interatrial and interventricular communication and after the ductus arteriosus ligation. It is rarely shown in this way in operated valve stenosis or under percutaneous, pulmonary or aortic interventions, except when the residual gradient is less than 10 mmHg, and even after the correction of the coarctation of the aorta. This scenario rarely appears after the correction of the total AV septal defect, as demonstrated in the present case. Among the corrected cyanogenic defects, two stand out with these same evolutionary characteristics, especially correcting the transposition of great arteries with the Jatene technique and anomalous pulmonary vein drainage.

**Conduct:** Even with this favorable evolution without residual defects and hemodynamic repercussions, purists still insist on antibiotic prophylaxis in these cases, which is very questionable, however. Such an attitude should rather be followed in the corrected patients, where residual defects still remain. Routine clinical follow-up, even in favorable cases, should be followed into adulthood considering the care required by the specialty itself.

**Comments:** The postoperative evolution of the total AV septal defect is marked by the usual presence of residual defects, especially of the AV valves, with little repercussion over time generally.^1^ It is estimated that this is due to abnormalities of the single AV valve that usually presents dysplasias, hypoplasias or redundancies as well as variations in the subvalvular apparatus, tendinous cords and papillary muscles.^2^ Another causal aspect of the valve problem is the change, at the time of surgical correction, of the valve elliptic morphology into a circular shape with the increased posterior circumference of the ring.^2^ The need for surgical re-intervention of the left AV valve is greater than the right valve, by approx. 5% and 3%, respectively.^3^ More discrete defects, which are common among operated patients, make it possible for them to have a healthy life generally. The evolutionary type described is rarely observed, with complete normalization of the anatomic-functional conditions.
